# Living with type 1 diabetes is challenging for Zambian adolescents: qualitative data on stress, coping with stress and quality of care and life

**DOI:** 10.1186/s12902-015-0013-6

**Published:** 2015-04-18

**Authors:** Given Hapunda, Amina Abubakar, Fons van de Vijver, Frans Pouwer

**Affiliations:** Department of Psychology, University of Zambia, P.O Box 32379, Lusaka, Zambia; Department of Culture Studies, Tilburg University, Tilburg, The Netherlands; Department of Medical and Clinical Psychology, Tilburg University, Tilburg, The Netherlands; Department of Psychology, Lancaster University, Lancaster, UK; Neuroassessment Unit, Centre for Geographic Medicine Research, KEMRI-WTRP, Kilifi, Kenya; Work Well Unit, North-West University, Potchefstroom, South Africa; School of Psychology, University of Queensland, Brisbane, Australia

**Keywords:** Diabetes, Adolescents, Stress, Quality of life, Zambia

## Abstract

**Background:**

Psychosocial problems are common in patients with diabetes. However, data on psychosocial issues affecting patients with diabetes in Zambia are scarce. The present study explored sources of stress, stress coping strategies, stigma and perceived quality of life and care as experienced by adolescents living with Type 1 Diabetes in Zambia.

**Methods:**

Semi-structured interviews were carried out. Three groups of participants involving adolescents with Type 1 Diabetes (n = 10), caregivers (n = 8) and health practitioners (n = 4) were interviewed. Transcripts were analyzed using a thematic approach.

**Results:**

Stress was commonly reported by adolescents mainly stemming from social, psychological and physical sources. To deal with stress, adolescents often employed different coping strategies such as adapting, accepting and avoiding among others. Both internal factors (those relating to the patients themselves) and external factors (those related to the context of the patients’) influenced the patients’ quality of health care. In addition, low quality of life was an issue among adolescents and their families. Poor diet, low socioeconomic status and lack of medicine were factors affecting quality of health care.

**Conclusion:**

Stress was an issue affecting adolescents; the coping strategies employed were sometimes maladaptive such as avoiding injecting themselves to escape stress. Several aspects of quality of life were suboptimal in both adolescents and their families, such as stigmatization, short life expectancy, low socioeconomic status and poor social participation. Findings show that there is an urgent need for a strong response from all stakeholders (governments, patients, organizations and companies) to improve diabetes care and living conditions for young people with type 1 diabetes living in Zambia.

## Background

Diabetes is one of the leading causes of mortality and disability around the world. The most common complications of diabetes that lead to mortality and disability include cardiovascular diseases, neuropathy, nephropathy, retinopathy and foot ulcers [1]. It is also common to find co-morbid problems among diabetes patients such as depression [2], hypertension [3], HIV and AIDS [4], and malaria especially in developing countries. To prevent acute and chronic complications of diabetes, treatment and care must be optimized by patients with diabetes and their health care team, for example by achieving and/or maintaining a good level of glycemic control.

Constantly seeking treatment and engaging in everyday self-care activities such as frequent glucose monitoring, following a meal plan, and correctly preparing or remembering to take insulin or oral medications at the right times can be a source of diabetes-specific emotional stress and can be difficult to follow a regime in times of stress for people with diabetes [5]. Stress refer to a physiological or a psychological response to external stimuli or to stressful events themselves which can be negative or positive or both [5]. Common signs of stress in patients include changes in sleep patterns, changes in appetite, anxious thoughts, and irritability [6]. General emotional stress can affect the blood glucose levels and glycemic control, and interfere with the ability to self-manage diabetes. Moreover it has been found to be associated with poor quality of life [5,7]. Stressful experiences influence diabetes control not only because of the devastating effect on poor blood glucose control but also because of the association between high blood glucose levels and the development of diabetes related complications [5,8]. For example, in prospective studies involving individuals with type 1 diabetes, patients who reported negative stress showed deteriorating glycemic control over time [9]. In addition to the physiological influence that stress has on glycemia, stress interferes with the ability to self-manage diabetes such as monitoring glucose frequently, following a meal plan and correctly preparing or remembering to take insulin or oral medication at the right time [5]. In adolescents with type 1 diabetes specifically, research shows that stress stems from the need to manage a complex medical condition that requires daily completion of multiple self-care behaviors, the impact of diabetes on social interactions with family members, peers and teachers as well as the interference of symptoms such as hypoglycemia with daily activities [10]. The observed effect of stress on individuals with diabetes shows great intra-individual and inter-individual variance depending on situational factors, type and amount of stress, personal characteristics and coping strategies [11]. The few African studies that have been conducted suggest that there is a link between stress and development of diabetes. In Kenya for example, patients linked diabetes to stress caused by disharmony, and conflicts within the family and strong emotions due to shock [12]. In South Africa, children with diabetes were found to have experienced more frequent stressful events compared to control children [13].

Chronic stress is an important risk factor for depression and depressed persons with diabetes have considerably lower Quality of Life (QoL) [14]. Most of the studies in this area have been conducted in Western countries, African data are still scarce. For instance in Zambia at baseline, mean values of QoL were significantly lower in adolescents with diabetes compared to health controls (19 vs. 22) [15]. In a Nigerian sample of 251 patients with diabetes, poor QoL was associated with diabetes-related physical complications, lower education status and having type 2 diabetes [16]. In Sub-Saharan countries, low QoL is exacerbated by inaccessibility of medical care and the relatively high costs of insulin, a lack of medical tools such as blood glucometers, poor infrastructure, inadequate training of health workers, and increased risk of misdiagnosis and failure to detect diabetes [17]. Given the above background, in the present study we explored sources of stress, ways of coping with stress, perceived quality of care and life as experienced by Zambian adolescents living with type 1 diabetes (T1D) from the viewpoints of the adolescents themselves, their caregivers and their health care providers. Specific objectives were to explore:

1. The adolescents’ sources of stress and the coping strategies that they used to deal with stress;
2. The perceived quality of health care and barriers to quality care for the adolescents;
3. The views of stakeholders on how diabetes affects the adolescents’ lives and their families.

## Methods

### Study location

Zambia is located in the southern part of Africa with an estimated population of 13,000,000 inhabitants. Zambia is categorized as a lower middle income nation [18]. Lusaka is the biggest city in Zambia with 2,198,996 inhabitants [19].

### Study site

The study was conducted at the Diabetes Clinic within the Pediatric Department of the University Teaching Hospital (UTH), the main referral hospital in Lusaka city. The Diabetes clinic serves approximately 109 diabetes patients (age range: newborns to 17 year olds). The services offered to them include medical reviews and examinations, such as blood glucose and ketone testing, treatment and disease management plans, diabetes education and whenever available, free supply of medical essentials, such as needles and syringes.

### Sampling procedure and sample size determination

Participants (adolescents, guardians of adolescents and health providers of adolescents with diabetes) were recruited using a purposive sampling method from the University Teaching Hospital in Lusaka. Adolescents with diabetes had to be aged between 12 and 18 years and currently using insulin therapy to meet inclusion criteria. Adolescents were included in the study because they were key informants on their experience living with diabetes. Guardians had to be primary caregivers (guardian here means any one responsible of taking care of the adolescent). It was important to include guardians because they had first-hand experience living with an adolescent with diabetes while health care practitioners had to work at the diabetes clinic to be involved in the study. Health care practitioners were included because they interacted with adolescents during clinical care; hence their views on experiences of adolescents with diabetes were valuable. 25 participants were approached but three (1 female guardian and an adolescent girl, and 1 male guardian) declined to participate in the study. The final sample size was 22, comprising 10 adolescents with T1D, 8 guardians of adolescents with T1D, and 4 health care practitioners (Table 1). We were able to reach interview data saturation from our adolescents’ interviews. However, because only a few guardians accompanied the adolescents during medical appointments we did not reach data saturation in the guardians; time and cost could not allow us to follow them up in their respective homes. Further, given a small number of health care providers who routinely attend to adolescents with diabetes, we interviewed all we could identify at the time of the study.

**Table 1:** **Demographic characteristics 1 of informants**

**Adolescents with Type 1 diabetes**		**N**	**%**
Sex	Males	2	20
	Females	8	80
Age			
	12-15 years (1 each)	4	40
	16 years	3	30
	17 years	3	30
Mean Age 15.3 years			
Grade	Grade 6	2	20
	Grade 8	2	20
	Grade 9	2	20
	Grade 10	2	20
	Grade 11-12	2	20
Duration on insulin			
	1 years	1	10
	2 years	2	20
	3 years	3	30
	5-6 years (1 each)	2	20
	7 years	2	20
Lives with			
	Biological parents	8	80
	Sibling (sister)	1	10
	Grandparent	1	10
**Guardians**		**N**	**%**
Sex			
	Males	2	25
	Females	6	75
Age	30-54 years	8	100
Mean age 37.3 years			
Number of children keeping			
	2 children	2	25
	4 children	2	25
	5 children	2	25
	7 children	2	25

### Data collection

The current study employed semi-structured interviews, because this qualitative methodology is particularly useful when researchers aim to understand the lived experiences, opinions, stories and views of the specific respondents on a certain phenomenon [20]. Interviews were conducted using open-ended questions. All interviews were held in a quiet place that was most convenient to participants and interviews were conducted by the first author (GH). The majority of interviews were conducted in English and five in either Bemba, Nyanja or Tonga (three local Bantu languages). The interviewer is a native Tonga speaker and individual multilingualism is common in Zambia. Interviews were audio recorded after permission of the participant and notes were also taken by the researcher. Interviews that were in the local languages were translated to English and all transcripts were checked by a research assistant who was fluent in the three local languages and English. Guiding questions focused on sources of stress, coping strategies and perceived quality of care and life as experienced by adolescents living with T1D. Semi-structured interviews were not piloted but they were discussed with health practitioners under the diabetes association for content-related validity (expert validity) before data was collected. The interviews lasted between 25 and 40 minutes.

**Table 2:** **Additional thematic quotations for adolescents with diabetes and caregivers of adolescents with diabetes**

**Theme**	**Source**	**Quotation**
**Stressors**	**Adolescents**	Sometimes my friends laugh at me because of the diet I take and I feel bad and most of the time am depressed. **Female, grade 11**
		In my family they want me to stop injecting myself. So what they say depresses me, they say that injecting myself is affecting my body. As for me I don’t feel good to injecting myself but it’s for my own good. **Female, grade 9**
		Too much, I feel low sometimes and I keep on asking the question “why me why me”. Sometimes I feel like I can stop and just develop a negative attitude towards diabetes. “How can I take it back, out of my life?” **Female, grade 12**
		I become depressed because of injecting myself, you see I have been injecting myself for seven years on the same spot but what can I do it’s my life. Most of my stress comes from injecting myself. **Female, grade 12**
		Because they want me to stop injecting myself and they also complain about transport is expensive and even the food for diabetes people is very expensive, it stresses me sometimes. **Female, grade 9**
		I worry that I be swollen again and my family worry about my health a lot especially when I am very ill like swollen. I can see when they are worried. They also worry about money, especially money for buying food and cloths. **Male, grade 8**
		Yah! Like when I am with friends they may buy sweet things (food) that I cannot eat and then the laugh at me. I can’t eat such food because I can get sick, so it’s very difficult and I feel depressed at times. **Female, grade 10.**
		I am stressed if the blood sugar is high. I start feeling very sick and sometimes vomiting and tired. I even get slim such that I start looking like I am HIV infected (Laughs). **Female, grade 10**
		I sit down most of the time and ask myself what made me to have diabetes, but I have to stop having such thought although I think a lot and I think I get stressed. **Female, grade 6**
		Sometimes I just ask why me? I have friends and most of them have no diabetes. But what I hate most is that I cannot take sweet things. And I also now wonder whether people with diabetes give birth, so I feel stressed. **Female, grade 10**
		I just feel feed up every time I have to inject myself take blood for sugar test and prick myself every time, so its stressful. **Female, grade 10**
		I worry about how it will affect my school or to find a job. Like other schools they refuse to take us, like one girl she also comes here and she was told to go back because she is sick (has diabetes-author insertion). **Female grade 8**
**Stress coping strategies**	**Adolescents**	Prayers! I pray a lot. Mostly alone but sometimes with my family members. I usually go for prayers on Tuesdays, Thursdays, Fridays, Saturdays and Sundays. **Male, grade 8**
		Sometimes when I am complaining, talking to my mother make me feel better. When I am stressed and depressed my friends cheer me up, they tell me fun stories and take me for a walk. They help me a lot. **Female, grade 12**
		I think knowing people who have diabetes and asking them how they have managed to live with diabetes can help me copy with stress. **Female, grade 10**
		I have to find what to do like at school I have to start playing with my friends that way I stop think about those things (stressors – author insertion). **Female, grade 11**
		I like to think of good things about my life, because I am free from any bad thoughts. **Female, grade 11**
		My friends tell me that I should be taking care of myself and not to eat sweet things (food- authors’ insertion) and a looking after myself and that makes me feel cared for. **Female, grade 9**
		I don’t usually get worried or stressed because I follow what I was told on how to inject myself with insulin. **Female, grade 6**
		I think if the hospital is near to me or may be if there is transport in place it will help me cope with the stress of transport money. **Female, grade 10**
		The bible, I sometimes just read the bible and pray to God to give me strength and just hala (call-authors’ insertion) my friends or my brothers will come and make up stories to make me laugh. **Female, grade 10**
		Sometimes it (stress) just comes like that; sometimes like when sugar is high, the Blood Pressure also goes up. And when I am feeling like that I just take water and sleep. **Female, grade 8**
**Stress coping strategies**	**Guardians**	The biggest thing that makes me less stressed is that when I compare to the way he was and now, I feel less burdened than when he was very sick. Right now, everything is seem fine I would not complain. But for others they are stressed because of lack of transport, but others its laziness like one parent here the doctors was scolding at her is a good example, her two children all have diabetes but she has never been bring them for reviews, that way when the problem is big they get more stressed. The children should be loved the way we love ourselves. Me this child is my grandchild and I have other children I am keeping they are all orphans but I make sure I take care of them and take them to hospital as if they are my own. **Female guardian aged 54**
		The big thing here is prayer, whenever I feel like praying I pray and it helps me. **Female guardian aged 35**
		It up to me I have to be ready that whenever she is in hypo, I can monitor and take her to the clinic. I have been given a letter to take but only when I suspect since I have no glucometer. But also to make sure that she has food when she is in hypos and make sure that she can be giving herself food when she suspects hypos**. Male guardian aged 37**
		Prayer, I just have to pray, whenever I few like praying, in the morning or afternoon I pray. **Female guardian aged 36**
		For me it is to pray and go to church. You see if you go to church you get a lot of encouragement and support that helps a lot to deal with her situation. **Female guardian aged 30**
**Diabetes care**	**Adolescents**	Umm, everything is ok, it’s just the place that makes me feel bad, the place makes me few like am very sick buts every thing is ok. **Male patient grade 8**
		Sometimes I come at the hospital and I find that there is no medicine, insulin, so they can be keeping more insulin and other medication. **Male, grade 8**
		They need to introduce classes on diabetes especially among youths. They need to be talking to us about things that we should not be indulging ourselves in. Just things, things that can worsen our health as youths. **Female, grade 12**
		If they could give out glucometers to everyone so that each one can be following the sugar levels. They should also start a gym for diabetes people. Like now I am gaining a lot of weight and it’s not good for my health but the gym could help me. **Female, grade 12**
		Most of us especially in my situation (with diabetes and poor-author insertion), we complain that transport is a problem coming this far (coming to the hospital–author insertion). **Female, grade 11**
		The biggest is distance and transport- you see we all have to come here (UTH) because in clinics they don’t do reviews. So we all have to come here for reviews and some come very far and transport is very expensive. **Female, grade 12**
		Also money for buying insulin is a problem, a bottle of insulin is US$10. In most cases we get insulin here but sometimes we have to buy. Mostly we have to get our own strips which cost at US$16. My father buys for me but sometimes he gets the pay late it’s difficult to get strips. **Female, grade 12**
		Us patients we complain about transport to come here for reviews but also many people do not have the money to pay for treatment. **Male, grade 7**

### Ethical approval

The study was approved by the ethics committee of the School of Humanities and Social Sciences, University of Zambia (reference number IRB: 00006464, IORG: 005376); all participants gave written informed consent.

### Data analysis

Audio recorded interviews were transcribed verbatim and translated to English whenever applicable and further checked for accuracy. In order to develop the themes and the categories, as near as possible to the material, an inductive category process was used [21]. After careful and thorough reading and re-reading, the initial coding and categorization of the themes was done by the first author and verified by the second author to ensure trustworthiness. Sentences or paragraphs related through their content or context were assigned codes and related codes were finally categorized as themes in a tally table. This process continued until no new themes emerged from the data. Emergent themes were discussed and refined by authors G.H and A.A. Data were analyzed manually.

## Results

**Figure 1:**
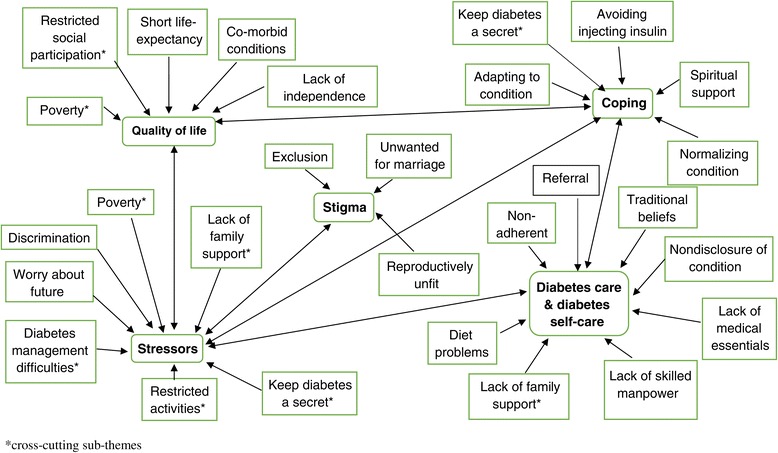
Thematic and sub-thematic mapping: possible 38 associations.

The study explored: stressors, stress coping strategies, diabetes care, quality of diabetes health care and life as experienced by young patients with diabetes in Zambia. Here we present data on the main forms of stressors in patients with diabetes which fall under social stressors (e.g. discrimination), psychological stressors (e.g. worry about the future) and physical stressors (e.g. poverty) and data on ways patients dealt with stressors to include: adapting to the stressor, avoiding the stressor, seeking spiritual support and normalizing the condition. Further factors that influenced patients’ perceptions of quality of health care are presented under the headings of internal factors (within control of patients) and external factors (outside the control of patients. The main issues that affected adolescents’ QoL and their families are presented as poor wellbeing, restricted social participation and perceived lack of independence. A thematic mapping to indicate possible associations among the themes identified is presented in Figure 1 and additional quotations on stress, stress coping strategies and diabetes care are available in the Table 2 attached to the manuscript.

### Stressors

About 18% [4/22] of the participants reported that adolescents experienced social stressors. An important social stressor in adolescents came from their need not to disclose their diabetes condition to others. Many adolescents worked hard to keep their condition a private issue. They were also stressed because of the discrimination they faced from others, mainly because peers thought diabetes is a communicable disease:

*Sometimes they face segregation, they let them play alone or let them do things alone because some of them think that it is communicable and they can get it if they hang out with them*. ***Pediatrician***

Another factor that contributed to social stress was lack of family support as exemplified by some family members pressuring the adolescents to stop injecting themselves insulin:

*In my family, they want me to stop injecting myself insulin. What they say stresses me a lot. They say that injecting myself is damaging my body.****Female, grade 9***

Psychological stressors was reported by 36% [8/22] participants and involved the feeling that the adolescents were different from other peers. Most adolescents were frustrated and wondered why they had to do certain things that their peers were not doing, such as carrying insulin and injection kits. Adolescents, especially the girls, were also stressed by the fact that the use of insulin was contributing to their weight gain but they still had to continue using it:

*Insulin makes them fat and when you look at adolescents particularly if I narrow it to the girls, they want to be slim.****Pediatrician***

Adolescents were also scared of diabetes related death which became a source of stress especially among the boys who thought having diabetes was a death sentence. There was also a worry especially among the female guardians and adolescents girls with diabetes that they were unwanted by men who think that they cannot bear a child:

*Children with such condition (diabetes) find it difficult to get married and cannot have children. Diabetes comes with many complications; so many men will not want to marry such a girl.****Female guardian 36 years old***

*Sometimes I just ask why me? I have friends and most of them have no diabetes. But what I hate most is that I cannot take (eat) sweet things (food – authors’ insertion). And I also now wonder whether people with diabetes give birth, so I feel stressed.****Female grade 10***

About 59% [13/22] of participants mentioned that adolescents face physical stressors. Physical stressors revolved around self-care activities including dietary limitations that adolescents faced because certain food stuffs (e.g. cakes and soft drinks) were not healthy and thus their intake was discouraged. In addition to diet restrictions, strict and rigid diabetes self-care was a source of stress especially the thought of injecting themselves multiple and painful insulin shots per day:

*Some inject themselves two times a day some three times a day. So the thing of injecting themselves every time is a stress as well. It brings stress to them whereby you have to be thinking about that needle and if you don’t do that anything can happen.****Diabetes Peer educator***

*I become depressed because of injecting myself, you see I have been injecting myself for seven years on the same spot but what can I do it’s my life. Most of my stress comes from injecting myself.****Female, grade 12***

Adolescents were stressed by the limitations they faced. For example, previously enjoyed activities were no longer possible due to the challenges of the danger of severe low blood sugar levels or of carrying medical essentials and also the worries of parents that adolescents may be unable to take care of themselves outside their parents’ homes. Many adolescents had challenges managing their sugar levels and experienced side effects of low or high sugar levels:

*I am stressed if the blood sugar is high I start feeling very sick and sometimes vomiting and become tired. I even get slim such that I start looking like I am HIV infected (Laughs).****Female grade 10***

For girls, having infections in their sexual organs (vaginal thrush) as a result of diabetes was stressful perhaps because they worried that they may have contracted sexually transmitted infections which are rampant among Zambian adolescents:

*Female adolescents often get stressed in the sense that they usually have vaginal thrush and that stresses them more because they think how did I get it especially if they do not engage in sexual relationships.****Pediatrician***

In addition, about 27% [6/22] of participants mentioned that adolescents experienced social stigmatization from peers and society especially among girls with diabetes who are seemingly not wanted for marriage. Social stigmatization was also common during play with peers. Adolescents were often excluded from play because others thought the condition was communicable:

*Sometimes my friends refuse playing with me, like my cousin used to say that we can’t play with you because you have a disease and we don’t want to catch it.****Girl, grade 9***

### Coping strategies

To cope with stress, about 14% [3/22] of participants mentioned that adolescents adapted to the situation, especially those that were diagnosed when they were still children, mainly because they were accustomed to having diabetes and managing it. Adolescents reframed the situation by thinking about their condition in a more positive way so as to get the best out of their health conditions. Other adolescents tried to change the situation through attending psychosocial support and psycho-educational activities:

*It also helps for instance through counselling like psychosocial support and not just educating them and giving support to both adolescents and their parents.****Diabetes Peer Educator***

Avoiding the stressor was also mentioned by about 14% [3/22] participants to be a coping strategy that adolescents used such as by avoiding injecting themselves insulin or engaging in activities that would distract them from stressful thoughts but also make them forget to take insulin:

*I have to find what to do. Like when I am at school I have to start playing with my friends that way, I stop thinking about those things (stressors – authors’ insertion).****Female, grade 11***

In addition, some adolescents accepted their diabetes by looking at the upside *“what does not kill you makes you stronger”*. Diabetes was looked at as an opportunity for personal growth. Seeking spiritual help through prayers was also a common 36% (8/22) strategy adolescents and their guardians used to cope with diabetes related stress:

*Prayers! I pray a lot. Mostly alone but sometimes with my family members. I usually go for prayers on Tuesdays, Thursdays, Fridays, Saturdays and Sundays.****Male, grade 8***

*For me it is to pray and go to church. You see if you go to church you get a lot of encouragement and support and that helps a lot to deal with her situation.****Female guardian aged 30***

Normalizing the situation so that the adolescents did not feel they were alone with their condition were other common coping strategies 10/22 (45%) among adolescents:

*We try to integrate them with other peers with diabetes so that they don’t feel they are alone in this situation. Once, they see their friends and know that they are also going through the same things as them it helps reduce the stress they go through.****Pediatrician***

### Diabetes care and diabetes self-care

Health care providers and guardians were the only ones who reported (83% [10/12]) that non-adherence to diabetes self-care activities and keeping diabetes a secret (which hindered help and care from others when adolescents were in need) were the main internally driven factors that affected the quality of care among adolescents. Non-adherent behaviors included not attending clinical appointments at the clinic, not changing site for injecting insulin, taking wrong dosages of insulin and avoiding taking insulin:

*Especially the girls when they reach adolescence they do not want to inject themselves with insulin in certain parts of the body like shoulder (upper arm -****authors’ insertion****) and thighs because these parts are important to a girl cosmetically but they are also good parts for insulin absorption. In addition insulin makes them fat, so they avoid it.****Nurse in Charge of Diabetes Clinic***

About 86% [19/22] of the participants also mentioned external factors as major problems for diabetes care and self-care. Externally driven factors that were reported to influence the quality of diabetes care included diet problems among adolescents because of poor economic background, and lack of medical commodities which could make some patients stay for days without insulin:

*Sometimes I come at the hospital and I find that there is no medicine, insulin, so they can be keeping more insulin and other medication.****Male, grade 8***

*Monitoring of sugar is practically impossible in our environment because when you look at a glucometer it is roughly about US$ 47.60 – US$ 65 and that just a one off thing because they need also strips which are expensive roughly about US$40 for 50 strips”.****Pediatrician***

Quality of care was affected by lack of family support in managing diabetes especially among young adolescents and traditional belief that certain herbs could cure diabetes:

*Others have their own beliefs that if you continue taking the medicine like insulin worsens the condition so they stop and start giving them (adolescents) herbal medicine.****Nurse in Charge of Diabetes Clinic***

Language limitations also affect quality of care because most Zambian languages have no equivalent words for some English diabetes and care-related words. As with many technical words, insulin does not feature in standard colloquial English any more than it does in any of the Zambian languages making it difficult to explain things:

*There are certain things that you cannot properly explain in a local language for instance when you say insulin, this person wants to understand what insulin is. Many times we don’t know the equivalent words in local languages, so language is a barrier.****Diabetes Peer educator***

In addition, lack of trained and specialized manpower to deal with diabetes cases especially diagnosing diabetes and delays in referrals. Data also revealed that respondents had the impression that diabetes was not yet among the priority diseases for authorities compared to malaria, HIV and AIDS. Co-morbid conditions also made it difficult to manage diabetes especially for children with malaria or HIV and AIDS. Specifically, adolescents and caregivers reported lack of nearby health facilities, expensive and sometimes inaccessible insulin, challenges handling medicine and following a recommended diet as some of the factors that were affecting quality of care. Others included poverty. Many patients bemoaned lack of money to buy food when they had an appointment at the hospital and they had to wait for a while before they could receive attention:

*The biggest is distance and transport- you see we all have to come here (UTH) because in clinics they don’t do reviews. So we all have to come here for reviews and some come very far and transport is very expensive. But also transport money and money for food. Especially if I am here and I am hungry I need money to buy the food, I was told to be eating but I cannot get money here.****Grade 8 male***

*The medicine that we receive requires that we keep it in the fridge but when I am at the farm I don’t know how I can handle this medication.****Female guardian aged 36***

### How diabetes affects adolescents’ QoL and their families

Diabetes was regarded as being associated with a shorter life expectancy due to various external factors including poor health care system, socioeconomic circumstances, lifestyle choices and unavailability of certain medical essentials:

*So really when you talk about an African child with diabetes the life span is quite short because of the health care system and the availability of the critical things that they need to survive on, that is the insulin. And also the commodities, the needles, the syringes are unavailable sometimes.****Pediatrician***

Other factors included poor socio-economic circumstances 86% [19/22], which hamper the use of advanced technologies to manage diabetes. Co-morbidity 14% [3/22], emotional 32% [7/22], cognitive 18% [4/22] and physical health problems 14% [3/22] were also issues that affected the QoL of adolescents and their families:

*My mother gets very emotional like she even cries and sometimes she tells me, “If I could take it away from you I would have done it”. But also sometimes when I am at school, I lose concentration and many times I can’t see properly on the blackboard.****Female grade 12***

The fact that having diabetes limited adolescents’ participation in social activities because their movements were restricted was mentioned by about 18% (4/22) as affecting their QoL. Many reported that they were expected not to stay away from their guardians’ homes for a long period of time in order for them to be monitored by adults:

*Sometimes if I want to do something or go somewhere they tell me, you have diabetes you don’t have to go somewhere, because one time I collapsed while at school because of hypoglycemic episodes and in the past I used to play with my friends for a long time, but now within a short period of time I have to go and eat.****Male in grade 8***

Adolescents’ sense of independence was mentioned by about 27% [6/22] as it seemed to have been adversely affected because of their condition. Many adolescents felt their guardians were constantly monitoring them. In addition, adolescents were worried about adjusting in their future roles without interference from others especially when experiencing hypoglycemia. This theme was mentioned about 55% [12/22] by our participants:

*My parents now have to monitor me and constantly ask me if I have injected or eaten. I feel bad.****Female, grade 10***

## Discussion

This study explored sources of stress, ways of coping with stress, perceived quality of care and life of Zambian adolescents living with T1D. To the best of our knowledge, this is the first qualitative study in Zambia to explore this subject providing views from all these three key stakeholders (adolescents, caregivers, and health professionals).

Having diabetes was regarded and experienced as an important source of stress in the present sample. Lack of support from family and others was reported to exacerbate stress in children and adolescents as they still depend on adults. The adolescents reported multiple sources of stress. There is not only an urgent need to help lower the stress levels experienced by adolescents, but also a strong need to educate family members. It was for example shocking to note that a family member gave potentially life endangering advice to a young person with T1D to stop injecting herself with insulin. Families can play a powerful role in the treatment of chronic illness not only by offering instrumental support but also by providing unconditional support and the opportunity for expression of emotions [22]. Evidence has shown that family functioning and stronger support are related to more optimal metabolic control in T1D [23]. Interventions involving joint sessions between adolescents and significant family members should be encouraged so that health care providers can give useful information about diabetes and also about the danger of certain traditional healing practices. They can also teach adequate skills that empower families in the process of not only adapting to the situation but also helping the adolescent manage the blood glucose levels and thus live a more fulfilling life with diabetes. However, family and other instrumental support systems are by far not enough; other sources of stress should be resolved through improving treatment and care, by increasing access to insulin, glucometers, strips and other basic medical essentials for patients with diabetes.

The adolescents in the present study used three main coping strategies: 1. “Adapting”, for example by accepting that one has diabetes. This coping style seemed more common in those who were diagnosed when they were still young; 2. “Normalizing”, such as assuring the adolescent patients they are not the only ones with diabetes; 3. “Avoiding” such as avoiding insulin injections or avoiding to talk about having diabetes. However, some of these strategies (notably avoidance) are not helpful and effective at least for long-term benefits; avoiding and engaging in distracting activities are short term strategies since most of the patients still come back to confront the stressor. In a Taiwanese sample of adolescents with T1D, avoidance was used when faced with unpleasant situations like increased inquisitiveness from peers about their conditions, leading to stopping self-management related behaviors in order to minimize attention from others [24]. Research shows that young patients with poor glycemic control are more likely to be stressed when they use maladaptive coping strategies [25]. The use of maladaptive coping strategies such as avoiding insulin injections is likely to contribute to exacerbation of their condition. Therefore, adolescents must be taught skills that are more adaptive such as improving self-care skills and healthy lifestyles coupled with positively interpreting their situations, doing something about the source of stress and accepting their health circumstances.

Almost a decade since the publication of a case study by Beran and colleagues [26] diabetes health care in Zambia is still sub-optimal. Patients still have difficulties acquiring insulin because of high costs and sometimes erratic availability. A study by the International Insulin Foundation in 2006 found that patients could only access insulin 26-49% of the time. Today the situation has not improved much according to the perceptions of adolescents, guardians and health care providers. The syringes used to cost between $0.01-$1.50 [27] but now only the patients in urban bigger hospitals are given free syringes whenever available, because sometimes the hospitals do run out of these commodities, whereas patients in rural areas still need to buy syringes. However, these commodities are not always available in hospitals and some patients stay for days without such medical essentials. Testing costs for outpatients used to be as high as $51.60 a month [26], now at least in urban big hospital testing is free because many patients cannot afford to buy their own glucometers. Patients still have problems accessing health care facilities because local clinics do not offer specialized diabetes care due to lack of adequate trained health care providers, leading to increased risk of misdiagnosis and late detection of diabetes. However, the Ministry of Health in Zambia now recognizes non-communicable diseases (NCD) as increasingly common conditions in the country and have since developed a strategic plan regarding NCD including diabetes. The NCD program has since embarked on a number of interventions for the prevention and early detection of NCDs. These include the development of treatment protocol, development of clinical nutrition and dietary guidelines, training of health workers in the management of NCDs, and raising awareness levels of NCDs among other [28]. Future studies should evaluate the impact of the strategic plan on the diabetes health-care system, especially commodity availability for patients, and needed health care providers and skills.

We found stigmatization and discrimination to be common experiences among our patients, particularly by peers and society at large. Peers often thought diabetes was contagious and thus avoided interacting with the patients while society had beliefs that diabetes patients were not reproductively fit and had low status value in romantic relationships especially among the girls. Similar beliefs were reported in Japan where both women and men with insulin dependent diabetes were 22% less likely to be married compared to 65% of their aged matched controls [29]. Stigma was practiced through restricting patients from engaging in most everyday activities, such as sports. The sources and causes of stigma in patients with diabetes differ across cultures. For instance, in Australia, stigma was perpetuated by the media, friends, health care professionals and teachers who often had a notion that diabetes was a self-inflicted condition and stigmatization often took the form of negative social judgments and exclusions from activities [30]. In Iran, persons with T1D were reported to be stigmatized as miserable humans, rejected marriage candidates and were deprived of a normal life because they were equated to disabled people who could not enjoy life [31]. Thus, it also seems important to educate family members and friends about diabetes in Zambia.

A systematic review on the QoL of children with T1D showed worry and treatment barriers, diabetes symptoms and worries, communication and treatment barriers, family burden related to diabetes, disease impact and disease related worries as the main issues that affect the quality of life in mainly Western children with T1D [32]. Our results were somewhat similar; however we found at least one more salient aspect in our sample: adolescents reported a limited sense of independence and social participation to be issues adversely affecting their lives; health care providers confirmed this perception. Being monitored by adults was one of the issues that affected adolescents’ sense of independence. Restricted and sometimes short play time were some of the issues that affect social participation and their lives. Improved treatment that can reduce dependence and perceived sense of intrusiveness from significant others such as the use of insulin pumps and care can improve the QoL of adolescents from developing countries. However insulin pumps are not available in Zambia because most patients cannot afford them. Our results are in sharp contrast with those obtained in developed countries, where on average, children with T1D show no difference in physical, psychosocial and overall quality of life as compared to their healthy peers [32].

Parental monitoring or support from significant others is essential for diabetes care in children and adolescents. Less parental monitoring is a risk for poor glycemic control [33]. However, monitoring and support should never be perceived as intrusive by the adolescent to avoid the impression that it affects their sense of independence. When adolescents perceive their parents to be intruding in their personal lives, they are likely to get rebellious which often leads to parent-adolescent conflict. It would therefore be important that parents of adolescents with diabetes learn to show support without risking a conflict. Families and parents must agree about diabetes management responsibilities, show supportive behaviors towards adolescents and collaborative problem solving plans, all of which have been shown to be associated with better regimen adherence and glycemic control [34].

This study had some limitations. To begin with, although the qualitative design enabled us to examine detailed experiences of the subjects in the study and to discover themes as well as their subjective views of the experience of living with diabetes, we cannot provide quantitative figures to underscore our findings, nor can we claim representativeness of our findings. Secondly we were unable to collect information from healthy peers to confirm the social stigmatization reported by our adolescents.

## Conclusion

In conclusion, lack of health care resources, stress and stigmatization, poor health care and inadequate advice by family and friends, due to a lack of knowledge are common problem in Zambian adolescents with diabetes. Adaptive strategies to buffer stress such as positive reframing and accepting of the condition, may be helpful to adolescents with diabetes. Major barriers are not only poverty and poor access to diabetes care, but also lack of knowledge among family members and friends. These barriers need to be removed in order to improve the quality of diabetes care and the quality of life of young persons with diabetes in Zambia. The themes related to stress, stigma, and diabetes care and self-care seemed interwoven, therefore solving some problems may have trickle down effects on other problems. Health authorities and policy makers in developing countries face the challenge of improving health care delivery to patients with T1D who also face high poverty levels. While therapies such as Continuous Subcutaneous Insulin Infusion (CSII) have shown robust improvements in glycemic control, reduced severe hypoglycemia and general QoL, only very few patients in Zambia can afford such therapies. Overcoming the economic barriers to providing good diabetes care in Zambian is still a formidable task.
